# A Combined CXCL10, CXCL8 and H-FABP Panel for the Staging of Human African Trypanosomiasis Patients

**DOI:** 10.1371/journal.pntd.0000459

**Published:** 2009-06-16

**Authors:** Alexandre Hainard, Natalia Tiberti, Xavier Robin, Veerle Lejon, Dieudonné Mumba Ngoyi, Enock Matovu, John Charles Enyaru, Catherine Fouda, Joseph Mathu Ndung'u, Frédérique Lisacek, Markus Müller, Natacha Turck, Jean-Charles Sanchez

**Affiliations:** 1 Biomedical Proteomics Research Group, Medical University Centre, Geneva, Switzerland; 2 Department of Parasitology, Institute of Tropical Medicine, Antwerp, Belgium; 3 Institut National de Recherche Biomedicale, Kinshasa, D.R. Congo; 4 Department of Veterinary Parasitology and Microbiology, Faculty of Science, Makerere University, Kampala, Uganda; 5 Department of Biochemistry, Faculty of Science, Makerere University, Kampala, Uganda; 6 Foundation for Innovative New Diagnostics (FIND), Geneva, Switzerland; 7 Swiss Institute of Bioinformatics, Medical University Centre, Geneva, Switzerland; New York University School of Medicine, United States of America

## Abstract

**Background:**

Human African trypanosomiasis (HAT), also known as sleeping sickness, is a parasitic tropical disease. It progresses from the first, haemolymphatic stage to a neurological second stage due to invasion of parasites into the central nervous system (CNS). As treatment depends on the stage of disease, there is a critical need for tools that efficiently discriminate the two stages of HAT. We hypothesized that markers of brain damage discovered by proteomic strategies and inflammation-related proteins could individually or in combination indicate the CNS invasion by the parasite.

**Methods:**

Cerebrospinal fluid (CSF) originated from parasitologically confirmed *Trypanosoma brucei gambiense* patients. Patients were staged on the basis of CSF white blood cell (WBC) count and presence of parasites in CSF. One hundred samples were analysed: 21 from stage 1 (no trypanosomes in CSF and ≤5 WBC/µL) and 79 from stage 2 (trypanosomes in CSF and/or >5 WBC/µL) patients. The concentration of H-FABP, GSTP-1 and S100β in CSF was measured by ELISA. The levels of thirteen inflammation-related proteins (IL-1ra, IL-1β, IL-6, IL-9, IL-10, G-CSF, VEGF, IFN-γ, TNF-α, CCL2, CCL4, CXCL8 and CXCL10) were determined by bead suspension arrays.

**Results:**

CXCL10 most accurately distinguished stage 1 and stage 2 patients, with a sensitivity of 84% and specificity of 100%. Rule Induction Like (RIL) analysis defined a panel characterized by CXCL10, CXCL8 and H-FABP that improved the detection of stage 2 patients to 97% sensitivity and 100% specificity.

**Conclusion:**

This study highlights the value of CXCL10 as a single biomarker for staging *T. b. gambiense*-infected HAT patients. Further combination of CXCL10 with H-FABP and CXCL8 results in a panel that efficiently rules in stage 2 HAT patients. As these molecules could potentially be markers of other CNS infections and disorders, these results should be validated in a larger multi-centric cohort including other inflammatory diseases such as cerebral malaria and active tuberculosis.

## Author Summary

The actual serological and parasitological tests used for the diagnosis of human African trypanosomiasis (HAT), also known as sleeping sickness, are not sensitive and specific enough. The card agglutination test for trypanosomiasis (CATT) assay, widely used for the diagnosis, is restricted to the *gambiense* form of the disease, and parasitological detection in the blood and cerebrospinal fluid (CSF) is often very difficult. Another very important problem is the difficulty of staging the disease, a crucial step in the decision of the treatment to be given. While eflornithine is difficult to administer, melarsoprol is highly toxic with incidences of reactive encephalopathy as high as 20%. Staging, which could be diagnosed as early (stage 1) or late (stage 2), relies on the examination of CSF for the presence of parasite and/or white blood cell (WBC) counting. However, the parasite is rarely found in CSF and WBC count is not standardised (cutoff set between 5 and 20 WBC per µL). In the present study, we hypothesized that an early detection of stage 2 patients with one or several proteins in association with clinical evaluation and WBC count would improve staging accuracy and allow more appropriate therapeutic interventions.

## Introduction

Human African trypanosomiasis (HAT), also called sleeping sickness, is a parasitic disease that occurs in sub-Saharan Africa. More than sixty million people are at risk of being infected. The World Health Organization (WHO) has reported impressive progress since 1995 in the control of HAT, leading to a substantial reduction of new cases detected yearly to 10'800 in 2007. The total number of cases is now estimated to be between 50'000 and 70'000 per year [1].

The parasite that causes HAT belongs to the *Trypanosoma brucei* family with two subspecies, *Trypanosoma brucei gambiense* and *Trypanosoma brucei rhodesiense*, responsible for the human disease. Trypanosomes are transmitted to humans by the bite of a tsetse fly and are initially confined to the blood, lymph nodes and peripheral tissues. This corresponds to the first stage (early stage; or haemolymphatic stage) of the disease. After an unknown period that varies from weeks to months, the parasites invade the central nervous system (CNS). This is called the second stage (late stage; or neurologic; or meningo-encephalitic stage) of HAT.

Clinical symptoms of HAT are not specific for the disease, and definite diagnosis is always based on parasitological examination of body fluids. The card agglutination test for trypanosomiasis (CATT), an assay that is based on trypanosome-specific antibody detection, is widely used for mass screening. However, it suffers from limited sensitivity and restricted to the *T. b. gambiense* form of the disease [2]. A positive parasitological diagnosis must always be followed by stage determination, which is performed by examination of the cerebrospinal fluid (CSF). This is a vital step in the diagnostic process, as the treatment differs depending on the stage of the disease. If HAT patients are not treated, they always die [3]–[5]. Early stage drugs are inefficient for late stage patients, and additionally, melarsoprol (MelB or Arsobal), which has been the most widely used drug to treat late stage patient, has itself an overall mortality rate of 5% due to its toxicity [6]. As a consequence, melarsoprol has in many countries been replaced by eflornithine as the first line treatment for *T. b. gambiense* infections but the latter drug suffers from important logistic constraints.

WHO defined late-stage HAT by the following criteria: presence of trypanosomes in CSF and/or an elevated WBC count above 5/µL of CSF [7]. However, presence of WBC in the CSF is not specific for the disease and parasite detection methods are not sensitive enough [8]. Furthermore, recent studies suggest the need to increase the cutoff between the first and second stages to 10 or 20 WBC/µL [2],[8],[9]. This has contributed to the concept of a potential intermediate stage of HAT with CSF WBC count >5 and ≤20 WBC/µL [10]. There is therefore a critical need for a reliable and efficient staging tool that would replace or complement trypanosome detection and WBC count.

Parasite migration and invasion of the CNS causes a neuroinflammatory process, associated with activation of microglial cells and astrocytes [11],[12], and infiltration of the CNS with leukocytes (predominantly mononuclear cells) [13]. Cytokines and chemokines are known to be actively involved in this process. Thus, TNF-α, IL-6, CXCL8 and IL-10 concentrations have been demonstrated to be elevated in the CSF of late-stage patients [11],[14] and the IFN-γ level has been reported as associated with the severity of the late stage disease [15]. The levels of CCL2, IL-1β and CXCL8 have also been correlated with presence of parasites in the CSF and neurological signs in HAT patients [16]. Additionally, levels of IL-1ra, G-CSF, VEGF, CCL4 and CXCL10 were found modulated in either the CSF or plasma of patients suffering from cerebral malaria [17]–[19], and could potentially be also modulated in HAT patients.

Proteomic analysis of human body fluids has become an important approach for biomarkers discovery [20]. In this context, we recently explored the concept of *post-mortem* CSF as a model of massive and global brain insult [21], which allowed the identification of potential brain damage biomarkers by proteomics strategies. Indeed, heart-fatty acid binding protein (H-FABP), identified from *post-mortem* CSF, has been validated as a marker of stroke [22] and Creutzfeldt-Jakob disease [23], respectively. Similarly, GSTP-1 was also found over-expressed in *post-mortem* CSF [24] compared to *ante-mortem*, and was recently validated as an early diagnostic marker of stroke and traumatic brain injury (Turck *et al*. Personal communication). Additionally, S100β protein has already been demonstrated to be a marker of blood-brain barrier (BBB) and neuronal damage [25] as well as a useful serum biomarker of CNS injury and a potential tool for predicting clinical outcome after brain damage [26].

In this context, we hypothesized that markers of brain damage discovered by proteomic strategies as well as inflammation-related proteins could individually or in combination indicate the CNS invasion by the trypanosome parasite. We measured the CSF concentrations of H-FABP, GSTP-1, S100β and thirteen inflammation-related proteins (IL-1ra, IL-1β, IL-6, IL-9, IL-10, G-CSF, VEGF, IFN-γ, TNF-α, CCL2, CCL4, CXCL8 and CXCL10) and evaluated their potential for staging the disease.

## Material and Methods

### Samples

Samples originated from a prospective observational study on shortening of post treatment follow-up in *gambiense* human African trypanosomiasis (THARSAT), conducted between 2005 and 2008 at Dipumba hospital in Mbuji-Mayi (Kasai Oriental province, Democratic Republic of the Congo). Details of the THARSAT study design and results are reported elsewhere (D. Mumba Ngoyi, in preparation). The study protocol was approved by the Ministry of Health, Kinshasa, DRC and by the Ethical Committee of the University of Antwerp, Belgium. Briefly, 360 *T. b. gambiense* patients in total were enrolled into the THARSAT study. Inclusion criteria were 1° confirmed presence of trypanosomes in lymph nodes, blood or CSF; 2°≥12 years old and; 3° living within a perimeter of 100 km around Mbuji-Mayi. Exclusion criteria were 1° pregnancy; 2° no guarantee for follow-up; 3° moribund; 4° haemorrhagic CSF before treatment and; 5° presence of another serious illness (active tuberculosis - treated or not, bacterial or cryptococcal meningitis). HIV and malaria were not considered as exclusion criteria. Each patient underwent a clinical examination. Staging of disease was based on CSF examination. WBC count was performed in disposable cell counting chambers (Uriglass, Menarini) and was performed in duplicate when the first count was <20 cells/µL. Trypanosomes were searched for in CSF by direct examination prior or during the cell counting procedure, followed by the modified single centrifugation method [27]. Second stage patients were defined as having >5 WBC/µL and/or trypanosomes in the CSF. First stage patients were defined as having 0–5 WBC/µL and no trypanosomes in the CSF. Patients having >5 and ≤20 WBC/µL and no trypanosomes in CSF were defined and treated as stage 2 patients, but highlighted as being in the potential intermediate stage. Patients or their responsible were informed about the study objectives and modalities and were asked to provide written consent. Treatment was provided according to the guidelines of the national control program for HAT (PNLTHA).

CSF samples were centrifuged immediately after collection. The supernatant remaining after the diagnostic procedure was aliquoted, stored and shipped frozen at −20°C or below. For the study reported here, a total of 100 CSF samples, taken before treatment, were tested. These samples originated from 21 stage 1 (S1) and 79 stage 2 patients (S2). S1 patients were age and sex matched with 21 S2 patients. Remainder S2 patients were chosen in order to obtain homogenous median age values. Patients were classified into three categories of neurological signs; absent (no neurological signs), moderate (at least one major neurological sign but no generalised tremors) or severe (at least two major neurological signs including generalised tremors). Major neurological signs were defined as: daytime somnolence, sensory and gait disturbances, presence of primitive reflexes (Babinski's sign, palmo-mental reflex, perioral reflex), modified tendon reflexes (exaggeration or abolition), abnormal movements such as tremor (fine, diffuse and generalised). Neurological signs were not reported for two patients.

### S100β, H-FABP and GSTP-1 measurements

The concentration of S100β was measured using a commercially available sandwich ELISA assay kit (Abnova, Taiwan) following the manufacturer's instructions. Briefly, calibrators, Quality control (QC) and CSF samples diluted 1∶4 were incubated 2 hours on microtiter strips pre-coated with polyclonal anti-cow S100β antibodies. After 3 washes, horseradish peroxidase (HRP) labelled anti-human S100β antibodies were added, incubated for 90 minutes and washed again before addition of the substrate solution (tetramethylbenzidine). Color development was stopped with sulphuric acid and absorbance was read on a Vmax Kinetic microplate reader, (Molecular Devices Corporation, Sunnyvale, CA, U.S.A.) at a wavelength of 450 nm.

H-FABP concentration was also determined using a commercially available ELISA kit (Hycult Biotechnology, Uden, Netherlands) according to the manufacturer's instructions. CSF samples (non-diluted) and standards were incubated (1 hour) together with peroxidase conjugated secondary antibodies in microtiter wells coated with antibodies recognizing human H-FABP. After 3 washes, tetramethylbenzidine was added and color development was stopped by adding citric acid.

The concentration of GSTP-1 was determined using a homemade ELISA as described by Allard *et al*. [28]. Briefly, biotinylated anti-GSTP-1 antibodies (2 µg/mL) (Biosite, California, USA) were coated onto a 96-well Reacti-Bind NeutrAvidin coated Black Plates (Pierce, Rockford, IL) for 1 hour at 37°C. After 3 washes, CSF samples (diluted 1∶4), quality controls and standards (recombinant GSTP-1 at concentrations ranging from 0 to 100 ng/mL) were incubated for 1 hour at 37°C, and followed by a washing step. Alkaline phosphatase conjugated antibodies against human GSTP-1 (Biosite, California, USA) at 2 µg/mL were added and incubated for 1 hour at 37°C. After 3 washes, Attophos AP fluorescent substrate (Promega, Madison, WI) was added and plates were read immediately on a SpectraMax GEMINI-XS (Molecular Devices Corporation, Sunnyvale, CA, U.S.A.) plate reader, using the kinetic mode. Vmax values were automatically calculated by the instruments based on relative fluorescence units (RFU) (λ_excitation_ = 444 nm and λ_emission_ = 555 nm).

Concentrations of S100β, H-FABP and GSTP-1 in the CSF samples were back-calculated using a linear calibration curve based on measured standards values.

### Bead suspension array

The levels of thirteen cytokines and chemokines (IL-1ra, IL-1β, IL-6, IL-9, IL-10, G-CSF, VEGF, IFN-γ, TNF-α, CCL2, CCL4, CXCL8 and CXCL10) were determined using the Bioplex bead suspension arrays according to the manufacturer's instructions (Bio-Rad, Hercules, CA). Briefly, thirteen sets of color-coded polystyrene beads were conjugated separately with one of the thirteen different antibodies against the molecule of interest. All the sets were then mixed together by the supplier and delivered ready-to-use. An equal amount of beads was added to each well of a 96-well filter plate. After a series of washes, standards and samples (diluted 1∶4) were added and incubated for 30 minutes at room temperature. After washing, a mix of the corresponding thirteen biotinylated detection antibodies was added and incubated 30 minutes at room temperature. After washing, streptavidin-phycoerythrin (streptavidin-PE) was added for 10 minutes. After a last series of washes, beads were re-suspended in the provided assay buffer and each well was aspirated using the Bio-Plex system. Each bead was identified and the corresponding target simultaneously quantified based respectively on bead color and fluorescence. The concentration of each target was automatically calculated by the Bio-Plex Manager software using corresponding standard curve (5-PL regression) obtained from recombinant protein standards.

### Data and statistical analysis

Descriptive statistics were performed using the SPSS (version 16.0, SPSS Inc., Chicago, IL, USA) and GraphPad Prism (version 4.03, GraphPad software Inc., San Diego, CA, USA) software. Because none of the markers presented a normal distribution in concentrations (Kolmogorov-Smirnov test), differences between groups were tested with non-parametric Mann-Whitney U test (comparison between two groups) and Kruskal-Wallis test followed by Dunn's post-hoc test (comparison between three groups). Statistical significance for these tests was set at 0.05 (2-tailed tests). The stage, the presence of the parasite in CSF and the severity of neurological signs were successively considered as the dependent variables. The different marker concentrations were considered as independent variables. Bivariate non-parametric correlations using the Spearman correlation coefficient were carried out with statistical significance set at 0.01 (2-tailed tests).

To calculate the sensitivity and specificity of each individual predictor with respect to staging, the specific receiver operator characteristic (ROC) curve of each analyte was determined and the cutoff value was selected as the threshold predicting stage 2 patients with 100% of specificity (Figure S1).

Aabel (version 2.4.2, Gigawiz Ltd. Co., Tulsa, OK, USA) was used for box plots, SPSS for scatter plots and R (version 2.8.0) [29] was used for plotting ROC curves.

### Panel development

Panel selection was mainly performed as described by Reynolds *et al*. [30]. Briefly, the optimized cutoff values were obtained by modified iterative permutation-response calculations (rule-induction-like, RIL) using only the molecules that presented a *p* value<0.0001 (Mann-Whitney U test), an AUC above 75% and a significant Spearman correlation with WBC above 0.4 (Table 2). Each cutoff value was changed iteratively by quantile of 2% increment and sensitivity was determined after each iteration until a maximum sensitivity was achieved for 100% specificity. The permutation–response calculations were conducted using a PERL program (ActivePerl version 5.10.0.1004, ActiveState Software Inc.) and data were coded in CSV format.

## Results

### Biomarker concentration as a function of disease stage

The main characteristics of the 100 patients evaluated in this study are presented in Table 1. The analytes were classified into three groups, based on the results presented in Table 2. Criteria for the classification were the significance (Mann-Whitney U test), the AUC and the correlation with WBC. In the first group (GR1) comprising IL-1ra, G-CSF, CCL4, and VEGF, no significant difference in CSF concentrations between the two stages of HAT was observed. The second group (GR2) encompassed IFN-γ, IL-9, CCL2 and S100β, for which concentrations in the CSF were significantly different between stage 1 and stage 2 patients (0.001<*p*<0.01, Mann-Whitney U test). The third group (GR3) included GSTP-1, H-FABP, TNF-α, IL-1β, IL-6, IL-10, CXCL8 and CXCL10, for which the difference between stages was highly significant (*p*<0.0001, Mann-Whitney U test) (Figure S2).

**Table 1:** Characteristics of the studied population.

		Stage 1	Stage 2
Population	n	21	79
Gender	Male	8	51
	Female	13	28
Age	Median (range)	32.0 (14–60)	33.0 (13–65)
WBC/µl	Median (range)	2 (0–5)	126 (6–6304)
Parasite in CSF	N	0	64
Neurological signs*	Absence	11	11
	Moderate	10	51
	Severe	0	15
>5 and ≤20 WBCµL No trypanosomes in CSF**	N	0	8

*: Neurological signs were not reported for two patients.

**: Correspond to the number of patients highlighted as being in the potential intermediate stage.

**Table 2:** Detailed results for all the molecules tested in respect with the stage of the disease.

		Absence of parasite and ≤5 WBC/µl	Presence of parasite and/or >5 WBC/µl	Mann-Whitney U test	Correlation with WBC	ROC curve		
	Markers	Median (range)	Median (range)	p value	(spearman rho)	% AUC	Cutoff [pg/mL]	Sensitivity, % (95% CI)^a^
GR3	CXCL10	347.3 (24.3–2048.8)	14130.0 (24.3–128900.0)	<0.0001	0.625**	95	>2080.0	84 (74–91)
	CXCL8	56.9 (1.3–96.5)	178.9 (1.6–1791.0)	<0.0001	0.557**	94	>97.1	82 (72–90)
	IL-10	6.7 (0.9–19.6)	74.5 (2.1–573.1)	<0.0001	0.702**	89	>20.0	80 (69–88)
	TNF-α	3.3 (0.5–8.4)	22.5 (1.0–295.4)	<0.0001	0.636**	93	>8.5	78 (68–87)
	H-FABP	226.4 (19.8–564.0)	748.3 (0.0–16680.0)	<0.0001	0.417**	86	>571.8	62 (50–73)
	IL-6	5.0 (0.2–57.7)	63.8 (0.8–3286.0)	<0.0001	0.732**	94	>58.0	52 (40–63)
	IL-1β	0.1 (0.1–0.7)	0.6 (0.1–42.2)	<0.0001	0.445**	80	>0.7	48 (37–60)
	GSTP-1	1272.9 (149.7–5026.9)	3014.0 (61.2–75810.0)	<0.0001	0.437**	79	>5078.0	24 (15–35)
GR2	IFN-γ	68.7 (8.6–209.2)	100.4 (1.7–995.5)	0.0049	0.094	70	>210.9	10 (4–19)
	IL-9	23.4 (3.6–44.5)	30.7 (3.6–209.6)	0.0051	0.041	70	>45.0	23 (14–34)
	S100β	43.2 (4.9–113.0)	78.4 (0.0–353.0)	0.0053	0.269**	70	>114.3	29 (19–40)
	CCL2	428.1 (58.6–632.9)	590.2 (15.8–5391.0)	0.0055	0.156	70	>664.7	44 (33–56)
GR1	G-CSF	43.4 (2.4–209.8)	63.2 (2.0–785.9)	0.0866 (ns)	−0.029	62	>281.7	4 (1–11)
	IL-1ra	817.3 (128.6–3087.6)	782.0 (34.0–11760.0)	0.5229 (ns)	−0.065	55	>3092.0	13 (6–22)
	CCL4	94.2 (1.5–301.0)	91.9 (5.4–753.9)	0.5423 (ns)	−0.143	54	>316.6	5 (1–12)
	VEGF	48.3 (20.0–215.7)	49.4 (3.5–1009.0)	0.9393 (ns)	−0.105	54	>222.4	9 (4–17)

a Sensitivity was set for a specificity of 100% (95% CI, 84–100).

**: Correlation is significant at the 0.01 level (2-tailed).

To assess the sensitivity and specificity of these analytes for S2 HAT, ROC curves were built. GR1 and GR2 had a low to medium area under ROC curve (AUC) ranging from 54 to 70% and also displayed a low sensitivity in detecting S2 patients (4–13% for GR1 and 10–44% for GR2, see Table 2) at a predefined specificity of 100%. GR3 showed higher AUC (79–95%), and sensitivities for identification of S2 patient up to 84% (Table 2). CXCL10 appeared then as the most accurate predictor for staging, as, with a cutoff set at 2080 pg/mL, this molecule identified 66 out of 79 late stage patients and ruled-out all the early-stage patients.

### Correlation between WBC and biomarker concentrations

**Figure 1:**
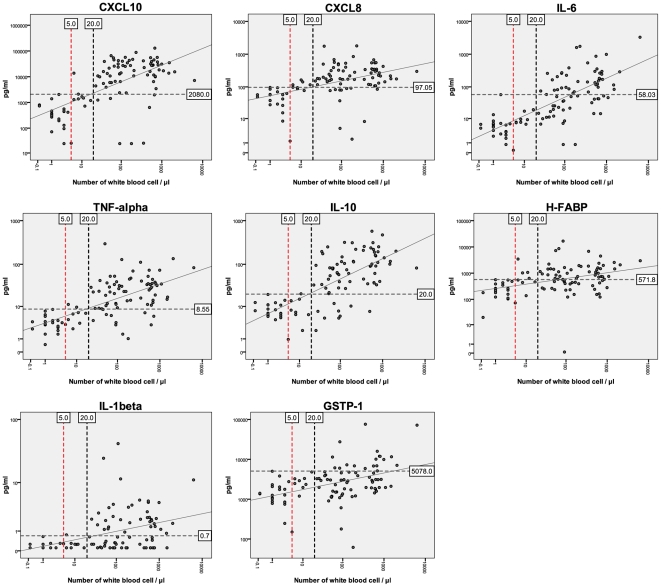
Scatter plots correlating the level of GR3 molecules with the WBC count. The horizontal dashed line corresponds to the cutoff value for the molecule that discriminates between S1 and S2 patients with a specificity of 100%. The left vertical dashed line corresponds to the WBC count cutoff value used for staging. The second vertical dashed line indicates the suggested cutoff value for staging. Patients between these lines (>5 and ≤20 WBC/µL) corresponded to potential intermediate stage patients. The diagonal line corresponds to the linear regression.

As the white blood cell count was one of the two reference staging parameters, we investigated the correlation between the concentrations of the sixteen biomarkers and the number of WBC in CSF (Table 2). There was no significant correlation in the concentrations of the first and second group of analytes (GR1 and GR2) with WBC, except for S100β, which had a significant but low Spearman rho coefficient (0.269, *p*<0.01). Otherwise, strong correlations were observed between WBC and the concentrations of GR3 biomarkers (GSTP-1, IL-1β, IL-6, H-FABP, TNF-α, IL-10, CXCL8 and CXCL10), with Spearman rho ranging from 0.417 to 0.732 (Table 2 and Figure 1). The levels of GR3 molecules in 8 potential intermediate stage patients (parasite not detected in CSF and having >5 and ≤20 WBC/µL) demonstrated the intermediate behaviour of this category with some patients appearing as S1 and others as S2 patients (Figure 1). Based on the above results, only the GR3 molecules (GSTP-1, IL-1β, IL-6, H-FABP, TNF-α, IL-10, CXCL8 and CXCL10) were selected for further analyses.

### Parasites in CNS and biomarker concentrations

**Figure 2:**
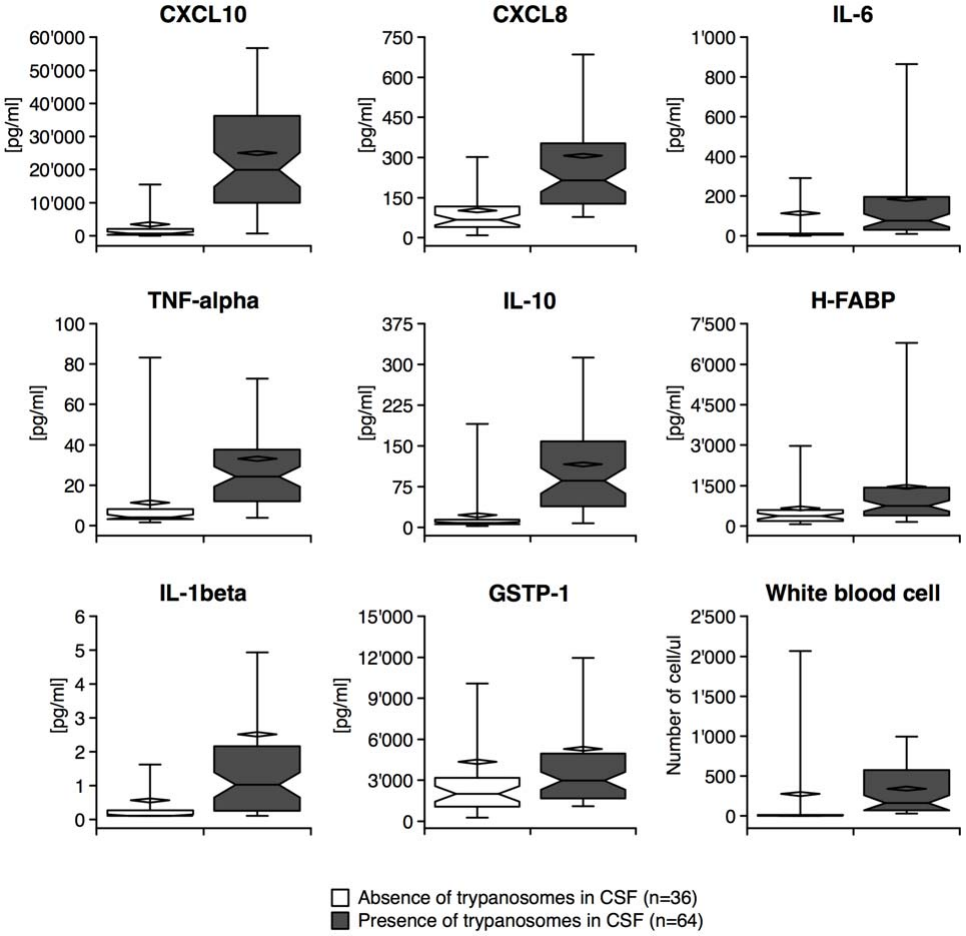
Box-plot of GR3 molecules and WBC classified according to the presence of the parasite in CSF. Median and mean are represented as a solid line in the box and a diamond respectively. Whisks are defined as 5^th^–95^th^ percentile without outliers. Half-width of the notch was calculated automatically by the software.

GR3 molecule concentrations were classified according to the absence/presence of trypanosomes in CSF. GSTP-1, IL-1β, IL-6, H-FABP, TNF-α, IL-10, CXCL8 and CXCL10 concentrations were significantly increased in patients with parasites in CSF (Figure 2 and Table S1). The six biomarkers associated with inflammation had a lower *p* value (<0.0001, Mann-Whitney U test) and higher AUC (ranging from 78% to 89%) than H-FABP and GSTP-1 (0.001<*p*<0.05, Mann-Whitney U test, AUCs of 69% and 64% respectively). Additionally, when only S2 patients were analysed, CXCL10, IL-10 and TNF-α levels still demonstrated a significant difference between patients with or without trypanosomes in CSF (*p*<0.05, Dunn's post-hoc test, Table S1).

### Neurological signs and biomarker concentrations

**Figure 3:**
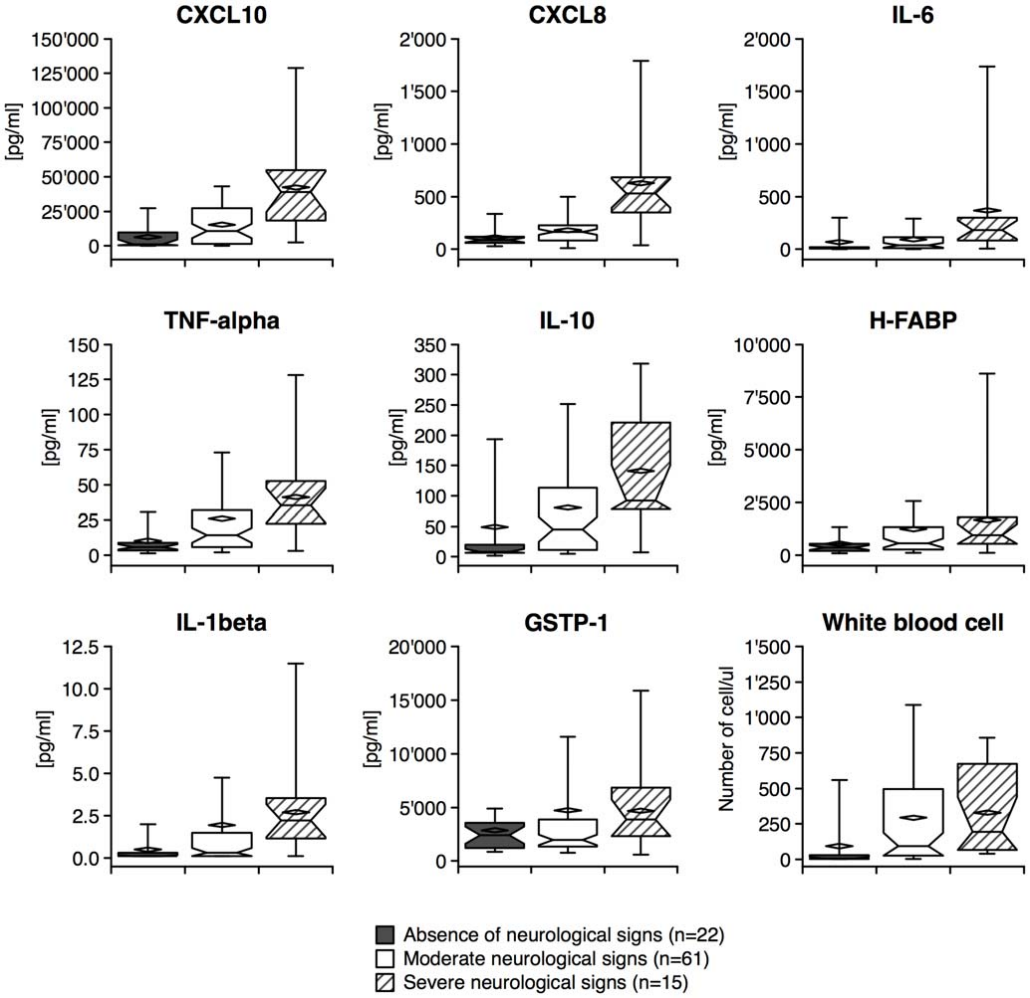
Box-plot of GR3 molecules and WBC classified according to the neurological signs. Median and mean are represented as a solid line in the box and a diamond respectively. Whisks are defined as 5^th^–95^th^ percentile without outliers. Half-width of the notch was calculated automatically by the software. Neurological signs of two patients were not reported (n = 98).

The patients were classified with respect to the neurological signs reported (absence, moderate or severe) (Figure 3). All the GR3 molecules except GSTP-1 showed a significant increase in concentration associated with higher severity of neurological signs (*p*<0.05, Kruskal-Wallis test). Indeed, CXCL10, CXCL8, IL-6, IL-10, IL-1β, and TNF-α concentrations were significantly different between patients without neurological signs and severe neurological signs (*p*<0.05, Dunn's post-hoc test), as well as between patients with moderate and severe neurological signs (*p*<0.05, Dunn's post-hoc test). H-FABP level was significantly different between patients without neurological signs and severe neurological signs (*p*<0.05, Dunn's post-hoc test). Only the concentrations of CXCL10, IL-10 and TNF-α could distinguish between absence and moderate neurological signs (*p*<0.05, Dunn's post-hoc test).

### Panel selection

In an effort to improve the global sensitivity of molecules in the prediction of second stage HAT, the GR3 molecules were combined using the rule induction like (RIL) approach. This resulted in the identification of a three-molecule panel characterized by CXCL10, CXCL8 and H-FABP (cutoff values were set at 2080.0, 97.1 and 571.8 pg/mL, respectively). A positive test (leading to identification of S2 patient) was obtained as soon as one of the three molecules included in the panel was above its cutoff value (Table 3). The panel had a sensitivity of 97% (95% CI, 91–100%) and, by definition, a specificity of 100% (95% CI, 84–100%). This means that the panel could identify 77 out of 79 stage 2 patients, and ruled-out all the 21 stage 1 patients. Out off the 77 ruled-in S2 patients, 5 were CXCL10 positive only (>2080.0 pg/mL), 6 CXCL8 positive only (>97.1 pg/mL) and 3 H-FABP positive only (>571.8 pg/mL). The rest of ruled-in S2 patients were identified with either 2 positive molecules (n = 23) or 3 positive molecules (n = 40). When this panel was applied on the intermediate stage patients (eight patients having >5 and ≤20 WBC/µL and no trypanosomes in CSF) only one patient gave a negative test response and thus 7 out of 8 patients were classified as S2.

**Table 3:** Detailed results for the three molecule panel in respect with the stage of the disease.

	Markers	Number of negative test	Number of positive test	Mann-Whitney U test , p value	% AUC (ROC curve)	Panel cutoff	Sensitivity, % (95% CI)^a^
Panel	CXCL10, CXCL8, H-FABP	23	77	<0.0001	99	≥1 molecule above its cutoff value^b^	97 (91–100)

a Sensitivity was set for a specificity of 100% (95% CI, 84–100).

b Cutoff values: CXCL10>2080.0 pg/mL, CXCL8>97.1 pg/mL and H-FABP>571.8 pg/mL.

## Discussion

In this study, including early and late stage HAT patients (n = 100), we evaluated sixteen molecules as potential staging markers of HAT, to replace or complement trypanosome detection and WBC count. Eight of these molecules, CXCL10, CXCL8, IL-6, IL-10, IL-1β, TNF-α, H-FABP and GSTP-1, presented concentrations significantly elevated in the CSF of late-stage HAT patients. We demonstrated that the CSF concentration of CXCL10 is highly elevated in stage 2 patients when compared to stage 1, highlighting this molecule as a potential new staging marker for sleeping sickness. A combinatorial approach has been applied in staging of HAT, in order to improve the sensitivity. This method has led to the identification of a panel consisting of CXCL10, CXCL8 and H-FABP, that identified late-stage patients with a sensitivity of 97% at 100% specificity.

H-FABP is a small protein belonging to the fatty acid-binding proteins (FABPs) and known to be expressed in the brain [31]. In myocardial infarction, HFABP is quickly released after the tissue damage [32],[33]. It has been suggested that the release of H-FABP from damaged cells could be used for diagnosis of acute and chronic brain injuries [31]. GSTP-1 is a member of the Glutathione S-transferase superfamily, playing a role in oxidative stress. Its expression in brain has not been well studied, but GSTP-1 seems to be the main isoform in brain [34] and may function as a brain damage biomarker [24]. Our results showed a higher level of both H-FABP and GSTP-1 in CSF of late stage patients compared to early stage patients. These two molecules are known to be associated with early brain cell death [24],[31], which could be correlated with the observed increase of their concentration in late-stage HAT patients. From now, it is not know if these two molecules were also associated with the inflammatory process.

Cytokines and chemokines play an important role in inflammatory processes and blood-brain barrier (BBB) dysfunction [35], and could therefore be potentially used as markers for staging HAT [11],[16],[36]. In the present study, the measured levels of inflammation-related proteins in CSF showed significant differences according to the disease progression. Indeed, concentrations of IL-1β, IL-6, IL-10, TNF-α, CXCL8 and CXCL10 were increased in the CSF of patients in late stage HAT compared to those in early stage of the disease. In addition, the levels of IL-1β, IL-6, CXCL8 and IL-10 were similar to those already reported for *T. b. gambiense* HAT [11],[16]. IL-1β is a pro-inflammatory cytokine that induces leukocytes infiltration [37] and is rapidly expressed in response to brain damage [35]. The high level of IL-1β found in CSF of stage 2 patients confirmed its probable association with the inflammatory process. Furthermore, its level was clearly correlated to the presence of severe neurological signs, supporting a potential release in relation to neurodegeneration. IL-6 and IL-10 are both anti-inflammatory cytokines. Their increased level in the CSF according to the stage as well as the severity of the neurological signs confirmed their activation associated with disease progression. The concentration of the two molecules was significantly increased in patients with more than 20 WBC/µL, which may suggest a probable expression after an already activated inflammatory process. Indeed, it has been demonstrated in vervet monkey models of HAT that IL-10 is associated with down-regulation of pro-inflammatory cytokines (IFN-γ and TNF-α) in the late stage of *T. b. rhodesiense* disease [38]. The level of the pro-inflammatory chemokine CXCL8 was also significantly elevated in CSF of S2 patients and correlated well with both presence of trypanosomes in CSF and severity of neurological signs. CXCL8 is a strong neutrophil attractant [16], which could thus not explain the good correlation of CXCL8 and the number of WBC (mainly B-lymphocytes) in CSF. However, its elevation in patients with a relatively low number of WBC (between 5 and 20/µL) suggests an early activation, which may play a role in BBB dysregulation [11].

The pro-inflammatory cytokine TNF-α has been reported as being involved in blood-brain barrier dysfunction [39]. These authors also demonstrated that trypanosomes may induce synthesis of TNF-α. In the present study, the increasing level of TNF-α was associated with disease progression as well as the presence of the parasite in CSF. These results suggested that parasites invasion into the CNS may lead to TNF-α production, which generated then CNS inflammation [14]. Additionally, an elevation according to the severity of the neurological symptoms was observed, which may support the neurotoxic effect of this cytokine in HAT [35].

CXCL10, also known as IP-10, is a pro-inflammatory chemokine with a central role in inflammatory responses [40]. The main effect of CXCL10 as a chemotactic molecule is activation of T cell migration to the site of inflammation, after binding to its receptor, CXCR3 [41]. The involvement of this chemokine in different CNS disorders has been demonstrated, such as viral meningitis [42] and multiple sclerosis [43], where increased CXCL10 levels in the CSF correlated with tissue infiltration of T lymphocytes [44]. In our study, the concentration of CXCL10 increased with progression of the disease, and was highly correlated with the number of WBC in CSF. Many studies have pointed out astrocytes as the primary source of CXCL10 at the level of the CNS and showed that this molecule is responsible, as chemoattractant, for the influx of activated T lymphocytes in brain [43], [45]–[47]. Indeed, there is a predominance of plasma cell infiltration in the brain of trypanosomiasis infected individuals. In addition, it has very recently been shown in a mouse model of HAT that CXCL10 may play an important role in T-cell recruitment into the brain parenchyma and is probably associated with brain invasion by trypanosomes [48]. Furthermore, the early activation of cytokine production (TNF-α, IL-6, and IFN-γ) by astrocytes and microglia in mice models infected with *T. brucei* before observation of an inflammatory response [49] has confirmed an important role of astrocyte activation in CNS inflammatory response. In consequence, early astrocyte activation, which induces CXCL10 production, is probably linked with BBB dysfunction and may occur before the inflammatory process. These hypotheses were supported by the increase CXCL10 concentration observed in patients having >5 and ≤20 WBC/µL but without trypanosomes detected in the CSF. The CXCL10 level was also demonstrated to be elevated in patients with cerebral malaria, and pointed out as potentially inducing apoptosis of endothelial cells leading to BBB breakdown [17] Recent work has suggested that neuronal apoptosis associated with calcium dysregulation may be induced by CXCL10 [50]. Even if mechanisms of CXCL10 mediated neurotoxicity remain unclear, we showed that the concentration of CXCL10 was correlated to the severity of neurological signs, supporting a possible involvement of this protein in neuronal injury pathways. Thus, CXCL10 expression in late stage HAT patients may be associated with both cell death and inflammatory process. Finally, active tuberculosis and pregnancy, two exclusion criteria in this study, have also been reported as modulating the level of CXCL10 [51],[52]. Although they have only been evaluated on serum and whole blood samples so far, it is not excluded that these criteria could potentially induce CXCL10 modulation in CSF. Nevertheless, our data demonstrated that CXCL10 is an efficient tool for staging patients, and suggested a potential role of CXCL10 as an early marker of parasite invasion into the CNS.

As the investigated proteins may be involved in different biological mechanisms, we evaluated in this study a strategy to combine results of each molecule, in order to find a panel able to discriminate more accurately early and late stage patients. This highlighted a panel of three molecules, including CXCL10 (the most promising single molecule), CXCL8 (another chemokine) and H-FABP (a marker of brain damage). With a specificity of 100%, this panel increased the sensitivity for staging of HAT patients up to 97% (compared to the 84% obtained with CXCL10 taken individually). Although the number of “intermediate” patients was small, the panel appeared to classify them rather as S2 patients (7/8 patients). This supports the current recommendation by WHO to consider such patients as S2 patients and treat them with drugs used for late stage disease. However, there is a need for more studies on *T. b. gambiense* and *T. b. rhodesiense* patients, before and after treatment, as well as on other parasitic diseases such as cerebral malaria, to verify these results and assess the feasibility of using the three-molecule panel as a complement to WBC count. There are obviously some drawbacks concerning this approach. Firstly the obtained panel is not 100% sensitive and thus some stage 2 patients will not be detected. The influence of other possible co-infections should also be evaluated in order to determine if they significantly modulate the evaluated molecules. Indeed, the three molecules included in the panel could potentially all be markers of other CNS disorders. It is also evident that the methods described in this study could not be implemented in such a way directly in the field and should be first transformed into a more simplified technique as for example a lateral flow immunoassay. Another limitation is the continued requirement of the invasive lumbar puncture since the molecules highlighted in this study have been evaluated on CSF samples.

In conclusion, the present study demonstrated the utility of inflammation-related proteins and brain damage markers as indicators of the second stage of HAT but potentially in other CNS disorders as well. We highlighted the value of CXCL10 as an efficient staging biomarker for *T. b. gambiense* infected HAT patients. Additionally, a combination of CXCL10 with CXCL8 and H-FABP resulted in a highly sensitive tool for identification of late stage HAT patients.

## Supporting Information

Figure S1 ROC curves of GR3 molecules and the panel. *Cut-off value for each molecule [pg/ml] and for the panel is displayed by a point and the numeric value. In parenthesis, sensitivity (%) of each molecule was set for 100% specificity. Area under the ROC curve (AUC) is also given.
(1.37 MB TIF)

Figure S2 Box-plot of GR3 molecules classified according to the stage of the disease. *Median and mean are represented as a solid line in the box and a diamond respectively. Whisks are defined as 5th–95th percentile without outliers. Half-width of the notch was calculated automatically by the software.
(0.69 MB TIF)

Table S1 Detailed results for GR3 molecules in function of the presence of trypanosomes in CSF (according or not to the stage) and the neurological signs.
(0.01 MB PDF)
