# Supplementary figures and images for: A Combined CXCL10, CXCL8 and H-FABP Panel for the Staging of Human African Trypanosomiasis Patients
Source: PLoS Negl Trop Dis. 2009 Jun 16;3(6):e459. doi: 10.1371/journal.pntd.0000459 (PMC2696178; doi:10.1371/journal.pntd.0000459)

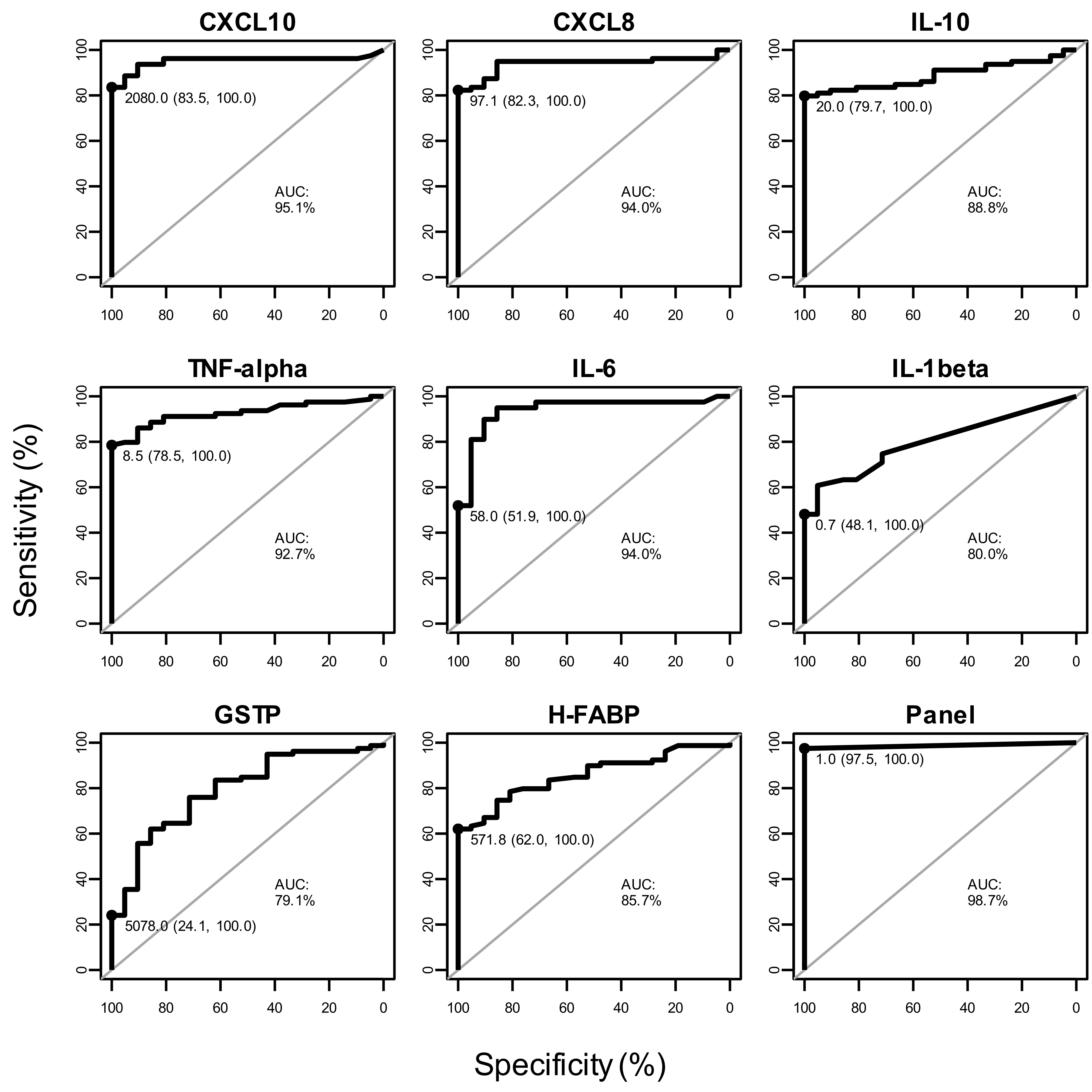

Supplement: Figure S1 — ROC curves of GR3 molecules and the panel. *Cut-off value for each molecule [pg/ml] and for the panel is displayed by a point and the numeric value. In parenthesis, sensitivity (%) of each molecule was set for 100% specificity. Area under the ROC curve (AUC) is also given. (1.37 MB TIF) [file pntd.0000459.s001.tif]

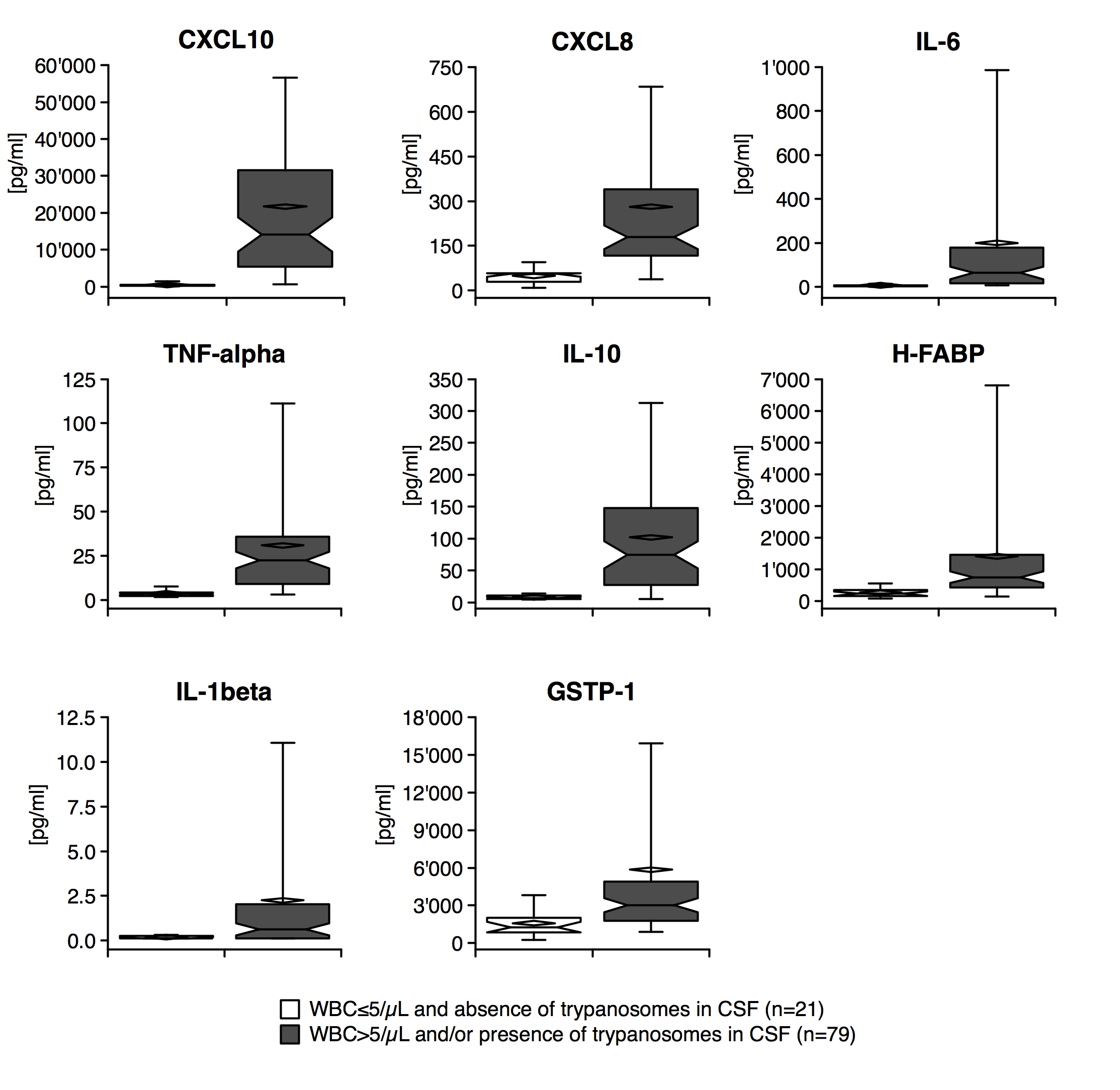

Supplement: Figure S2 — Box-plot of GR3 molecules classified according to the stage of the disease. *Median and mean are represented as a solid line in the box and a diamond respectively. Whisks are defined as 5th–95th percentile without outliers. Half-width of the notch was calculated automatically by the software. (0.69 MB TIF) [file pntd.0000459.s002.tif]
