# Supplementary material for: A Combined CXCL10, CXCL8 and H-FABP Panel for the Staging of Human African Trypanosomiasis Patients
Source: PLoS Negl Trop Dis. 2009 Jun 16;3(6):e459. doi: 10.1371/journal.pntd.0000459 (PMC2696178; doi:10.1371/journal.pntd.0000459)
Supplement: Table S1 — Detailed results for GR3 molecules in function of the presence of trypanosomes in CSF (according or not to the stage) and the neurological signs. (0.01 MB PDF) [file pntd.0000459.s003.pdf]

Table S1: Detailed results for GR3 molecules in function of the presence of trypanosomes in CSF (according or not to the stage) and the neurological signs.

**Trypanosomes in CSF**		Without<br>Median (range)	With<br>Median (range)	Mann-whitney<br>p value	ROC curve<br>% AUC	 CO [pg/ml]	 Sensitivity % (95% CI)*
GR3	CXCL10	727.1 (24.3-44930.0)	19980.0 (24.3-128900.0)	<0.0001	88	> 11699.0	67 (54-78)
	CXCL8	68.8 (1.3-545.5)	215.0 (1.6-1791.0)	<0.0001	83	> 206.4	52 (39-64)
	IL-10	7.9 (0.9-200.1)	86.3 (2.1-573.1)	<0.0001	87	> 37.0	77 (64-86)
	TNF-a	4.1 (0.5-134.7)	24.3 (1.0-295.4)	<0.0001	86	> 11.2	78 (66-87)
	IL-6	6.8 (0.2-3)	77.0 (0.8-1736.0)	<0.0001	86	> 106.4	41 (29-54)
	IL-1b	0.1 (0.1-11.1)	1.0 (0.1-42.2)	<0.0001	78	> 0.8	53 (40-66)
	GSTP1	2000.0 (149.7-71820.0)	2962.0 (61.2-75810.0)	0.0218	64	> 5078.0	25 (15-37)
	HFABP	368.4 (19.8-3451.0)	747.7 (0.0-16680.0)	0.0018	69	> 2149.0	11 (5-21)
	WBC	3.0 (0.0-6304.0)	163.5 (23.0-1521.0)	<0.0001	89	> 635.5	20 (11-32)

* sensitivity was set for a specificity of 92% (95% CI, 78-98)

**Trypanosomes in CSF**<br>(according to the stage)		WBC<5/ul, no trypa (1)<br>Median (range)	WBC>5/ul, no trypa (2)<br>Median (range)	WBC>5/ul, Trypa (3)<br>Median (range)	Kruskal-wallis<br>p value	Dunn's Multiple Comparison Test<br>1 Vs 2	 1 Vs 3	 2 Vs 3
GR3	CXCL10	347.3 (24.3-2049.0)	2388.0 (722.5-44930.0)	19980.0 (24.3-128900.0)	<0.0001	< 0.05	< 0.001	< 0.05
	CXCL8	56.9 (1.3-96.5)	142.2 (37.3-545.5)	215.0 (1.6-1791.0)	<0.0001	< 0.01	< 0.001	ns (> 0.05)
	IL-10	6.7 (0.9-19.6)	13.9 (2.1-200.1)	86.3 (2.1-573.1)	<0.0001	ns (> 0.05)	< 0.001	< 0.01
	TNF-a	3.3 (0.5-8.4)	8.7 (2.4-134.7)	24.3 (1.0-295.4)	<0.0001	< 0.05	< 0.001	< 0.05
	IL-6	5.0 (0.2-57.7)	11.0 (6.4-3286.0)	77.0 (0.8-1736.0)	<0.0001	< 0.05	< 0.001	ns (> 0.05)
	IL-1b	0.1 (0.1-0.7)	0.3 (0.1-11.1)	1.1 (0.1-42.2)	<0.0001	ns (> 0.05)	< 0.001	ns (> 0.05)
	GSTP1	1273.0 (149.7-5027.0)	3120.0 (609.4-71820.0)	2962.0 (61.2-75810.0)	0.0002	< 0.01	< 0.001	ns (> 0.05)
	HFABP	226.4 (19.8-564.1)	863.4 (138.3-3451.0)	747.7 (0.0-16680.0)	<0.0001	< 0.001	< 0.001	ns (> 0.05)

**Neurological signs**		Absence<br>Median (range)	Moderate<br>Median (range)	Severe<br>Median (range)	Kruskal-wallis<br>p value	Dunn's Multiple Comparison Test<br>Absence vs Moderate	 Absence vs Severe	 Moderate vs Severe
GR3	CXCL10	1243.0 (25.4-37570.0)	10770.0 (24.3-65600.0)	39160.0 (2388.0-128900.0)	<0.0001	< 0.05	< 0.001	< 0.01
	CXCL8	86.4 (1.3-402.1)	163.9 (1.6-674.7)	531.9 (37.3-1791.0)	<0.0001	ns (>0.05)	< 0.001	< 0.001
	IL-10	8.4 (0.9-573.1)	45.0 (2.1-471.5)	92.5 (7.6-318.3)	0.0002	< 0.05	< 0.001	< 0.05
	TNF-a	6.0 (0.5-72.9)	14.2 (1.0-295.4)	35.4 (3.2-128.3)	<0.0001	< 0.05	< 0.001	< 0.05
	IL-6	8.7 (0.2-865.7)	37.1 (0.8-986.4)	183.4 (6.9-1736.0)	<0.0001	ns (> 0.05)	< 0.001	< 0.01
	IL-1b	0.1 (0.1-4.5)	0.3 (0.1-42.1)	2.2 (0.1-11.5)	<0.0001	ns (> 0.05)	< 0.001	< 0.01
	GSTP1	2411.0 (149.7-11980.0)	1971.0 (61.2-75810.0)	3881.0 (609.4-15910.0)	0.1826 (ns)	ns (> 0.05)	ns (> 0.05)	ns (> 0.05)
	HFABP	368.4 (72.9-3451.0)	579.4 (0.0-16680.0)	963.2 (122.5-8622.0)	0.0127	ns (> 0.05)	< 0.05	ns (> 0.05)
	WBC	5.5 (1.0-778.0)	92.0 (0.0-2064.0)	192.0 (38.0-856.0)	0.0002	< 0.01	< 0.001	ns (> 0.05)
